# Transcriptomic and Proteomic Analysis of Mannitol-metabolism-associated Genes in *Saccharina japonica*

**DOI:** 10.1016/j.gpb.2018.12.012

**Published:** 2020-11-25

**Authors:** Shan Chi, Guoliang Wang, Tao Liu, Xumin Wang, Cui Liu, Yuemei Jin, Hongxin Yin, Xin Xu, Jun Yu

**Affiliations:** 1 College of Marine Life Sciences, Ocean University of China, Qingdao 266003, China; 2 Qingdao Haida BlueTek Biotechnology Co., Ltd., Qingdao 266003, China; 3 CAS Key Laboratory of Genome Sciences and Information, Beijing Key Laboratory of Genome and Precision Medicine Technologies, Beijing Institute of Genomics, Chinese Academy of Sciences, Beijing 100101, China; 4 University of Chinese Academy of Sciences, Beijing 100049, China; 5 Beijing Key Laboratory of Agricultural Genetic Resources and Biotechnology, Beijing Agro-Biotechnology Research Center, Beijing Academy of Agriculture and Forestry Science, Beijing 100097, China; 6 College of Life Sciences, Yantai University, Yantai 264005, China

**Keywords:** Mannitol metabolism, *Saccharina japonica*, RNA-seq, Proteomic analyses, Biochemical characterization

## Abstract

As a carbon-storage compound and osmoprotectant in brown algae, mannitol is synthesized and then accumulated at high levels in ***Saccharina japonica*** (*Sja*); however, the underlying control mechanisms have not been studied. Our analysis of genomic and transcriptomic data from *Sja* shows that **mannitol metabolism** is a cyclic pathway composed of four distinct steps. A mannitol-1-phosphate dehydrogenase (M1PDH2) and two mannitol-1-phosphatases (M1Pase1 and MIPase2) work together or in combination to exhibit full enzymatic properties. Based on comprehensive transcriptomic data from different tissues, generations, and sexes as well as under different stress conditions, coupled with droplet digital PCR (ddPCR) and proteomic confirmation, we suggest that *SjaM1Pase1* plays a major role in mannitol biosynthesis and that the basic mannitol anabolism and the carbohydrate pool dynamics are responsible for carbon storage and anti-stress mechanism. Our proteomic data indicate that mannitol metabolism remains constant during diurnal cycle in *Sja*. In addition, we discover that mannitol-metabolism-associated (MMA) genes show differential expression between the multicellular filamentous (gametophyte) and large parenchymal thallus (sporophyte) generations and respond differentially to environmental stresses, such as hyposaline and hyperthermia conditions. Our results indicate that the ecophysiological significance of such differentially expressed genes may be attributable to the evolution of heteromorphic generations (filamentous and thallus) and environmental adaptation of Laminariales.

## Introduction

Mannitol is one of the most common sugar alcohols in nature and is produced by a variety of living organisms, including bacteria, fungi, terrestrial plants, and algae [1], [2]. The presence of mannitol has been reported in primary endosymbiotic algae (*e.g.*, species belonging to Chlorophyta [3], [4] and a few species of Rhodophyta [5], [6]), as well as in secondary endosymbiotic Ochrophyta algae (*e.g.*, brown algae [7], [8], [9]) and some other stramenopiles (*e.g.*, *Olisthodiscus* sp. [10]). As one of the primary photosynthetic products and storage compounds in Laminariales [11], [12], [13], mannitol can be stored up to 15%–26% of the organism’s dry weight [14], [15]. Moreover, mannitol fulfills key physiological roles, including protecting against environmental stresses and acting as an organic osmolyte, compatible solute, antioxidant, or thermal protectant [1], [16], [17]. Despite the physiological importance of mannitol in brown algae, genes involved in mannitol biosynthesis have been characterized only in *Ectocarpus* but not in kelps [18], and only a few genes’ functions in this biosynthetic pathway have been confirmed. Moreover, the regulatory mechanisms are yet to be understood.

*Saccharina japonica* (*Sja*) is one of the most important brown macroalgae in the order Laminariales because of its considerably high biomass and economic significance [19]. Asian countries have been cultivating the *Sja* species since early 1950s [20], and presently, its annual commercial production (7.7 million tons) ranks the second highest among all aquacultural species [21]. Moreover, purified compounds derived from *Sja*, such as mannitol, have been widely used as food supplements, medicines, and chemical materials for chemical industry and scientific research [22], [23], [24], [25]. The *Sja* life cycle comprises three stages, including the single-cell (meiospore), multicellular filamentous (gametophyte, *n*), and large parenchymal individual (sporophyte, 2*n*) stages [19]. The unique heteromorphic alternation of generations of *Sja* makes it different from its close relatives in the genus *Ectocarpus* that lack the parenchymal stage [26]. Brown algae are the only secondary endosymbiotic taxa in which multicellularity has evolved [26], [27], [28], but the regulatory mechanisms responsible for the structural difference between filamentous brown algae (*Ectocarpus*) and heteromorphic haploid-diploid algae (*Saccharina*) are not yet well understood. Moreover, Laminariales, such as *Sja*, are predominant in the marine ecosystems of cold and temperate coastal zones with harsh and extreme climates [29].

The availability of the *Ectocarpus siliculosus* genome has paved the way for studying the molecular basis of mannitol biosynthesis in algae [30]. The biosynthesis involves two enzymatic steps: the first step is the reduction of fructose-6-phosphate (F6P) to mannitol-1-phosphate (M1P) by mannitol-1-P dehydrogenase (M1PDH; EC 1.1.1.17), and the second step is the hydrolysis of M1P to mannitol by mannitol-1-phosphatase (M1Pase; EC 3.1.3.22) [1]. A recent analysis of mannitol biosynthesis revealed mannitol-metabolism-associated (MMA) genes in algae. For example, MMA genes encoding M1PDHs and haloacid dehalogenases (HAD-M1Pases) have been found in the members of Phaeophyceae, including Ectocarpales and Laminariales [17], [31], [32]. Previous phylogenetic analyses suggested that these genes were imported into brown algae by horizontal gene transfer from Actinobacteria [18]. Subsequently, a more comprehensive assessment across various algal lineages confirmed that these genes may be present in nonphotosynthetic eukaryotic host cells involved in endosymbiosis [17]. Native M1PDH and M1Pase activities have previously been characterized in cell-free extracts from red algae *Dixoniella grisea* [6], *Caloglossa continua* [33], [34], and *Caloglossa leprieurii* [5], brown algae *Spatoglossum pacificum*, *Dictyota dichotoma*, and *Laminaria digitata* [35], [36]), and green alga *Platymonas subcordiformis* [37]; however, genes encoding these enzymes have not been identified. In the model brown alga *E. siliculosus*, recombinant EsM1PDH1cat (containing only the catalytic domain) and EsM1Pase2 have been characterized [2], [32], [38]. Determination of kinetic parameters indicated that EsM1PDH1cat exhibited higher catalytic efficiency for F6P reduction than M1P oxidation; EsM1Pase2 was shown to hydrolyze the phosphate group from M1P to produce mannitol, but it was inactive on hexose monophosphates such as glucose-1-phosphate (G1P), glucose-6-phosphate (G6P), and F6P [2], [32]. Moreover, gene expression analysis showed that transcription of three *M1PDHs* and two *M1Pases* in *E. siliculosus* (a filamentous brown alga) was influenced by the diurnal cycle, and *EsM1Pase1* was highly down-regulated under hyposaline stress [32]. However, these genes remain poorly illustrated in *Sja* (a large parenchymal brown alga).

In this study, we systematically characterized the *M1PDH* and *M1Pase* genes and their encoded proteins, which are known to act in the *Sja* mannitol biosynthesis, based on transcriptomic and proteomic data generated from different *Sja* tissues of various generations (including the sporophyte and gametophyte generations) or under different abiotic stress conditions. Our results extend the understanding of mannitol metabolic pathways and their regulatory mechanisms in the context of ecophysiological and evolutionary significance of Laminariales. In addition, *Sja* MMA genes may be used to engineer microbes for mannitol production, and also to engineer plants for increasing their tolerance to abiotic stresses and for mannitol biosynthesis for subsequent extraction.

## Results

### A combined analysis of RNA-seq and Tandem Mass Tag

**Figure 1:**
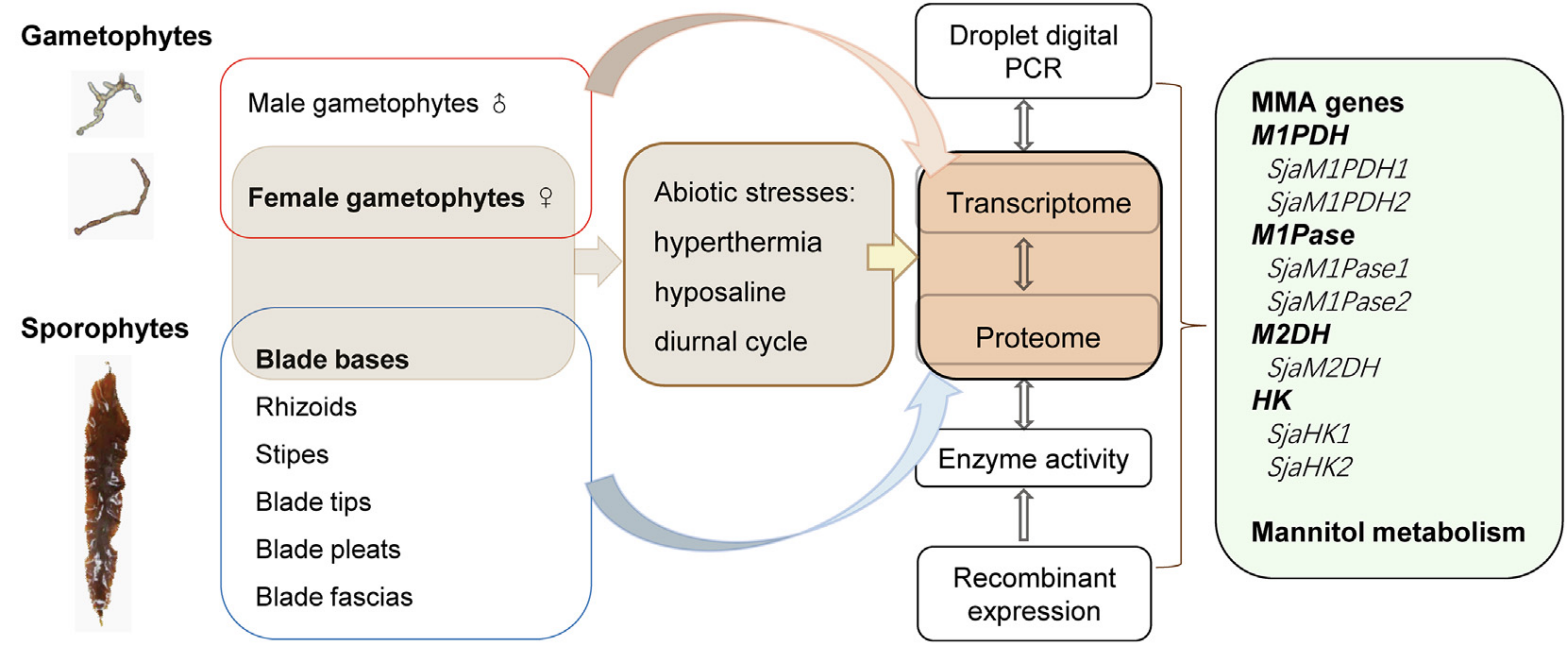
**A flow chart of the studydesign** Twelve *Saccharina japonica* (*Sja*) samples were collected for transcriptome sequencing, including two separately from male and female gametophytes, six from different parts of thalli (rhizoids, stipes, blade tips, blade pleats, blade bases, and blade fascias), and four from *Sja* incubated under different abiotic stresses (hyperthermia and hyposaline). Four samples from female gametophytes incubated under different conditions (*i.e.*, control, hyperthermia, hyposaline, and darkness) were collected for proteome sequencing. Droplet digital PCR (ddPCR) was used to confirm the RNA-seq results. All mannitol-metabolism-associated (MMA) genes from *Sja* were annotated based on transcriptomic and proteomic data. Some native MMA genes were over-expressed in *Escherichia coli* to characterize their enzyme activities.

**Supplementary Figure 1:**
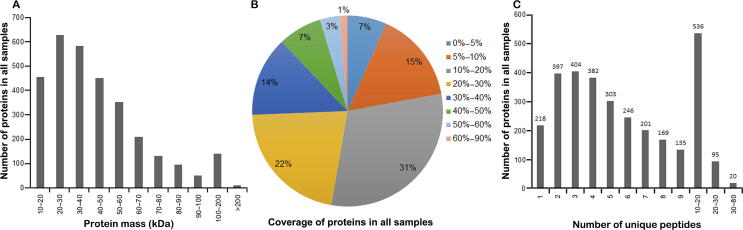
**General information about the proteomic analysis of the algal samples A.** Distribution of proteins of different molecular weights. **B.** Coverage of proteins obtained by using TMT LC-MS/MS-identified peptides. 93% of these proteins have a coverage that was >5%. **C.** Number of unique peptides of the detected proteins. 93% (2888) were inferred from at least two unique peptides.

Our combined analysis started with transcriptome sequencing of 12 *Sja* samples, including two samples separately from male and female gametophytes, six from different parts of thalli (rhizoids, stipes, blade tips, blade pleats, blade bases, and blade fascias), and four from *Sja* incubated under different abiotic stresses (hyperthermia and hyposaline conditions) (Figure 1). Approximately 9 Gb per algal sample and a total of 336 Gb of RNA-seq raw data were acquired (Table 1). After removing low-quality (< Q20) reads and trimming, the clean reads ranged between 95.21% and 96.70% of the raw reads, and the GC contents ranged from 53.79% to 57.11%. Our Tandem Mass Tag (TMT) proteomic analysis identified a total of 3106 proteins, using samples from female gametophytes incubated under different conditions (*i.e.*, control, hyperthermia, hyposaline, and darkness). The molecular weight of most identified proteins ranged from 20 kDa to 70 kDa (71%, Figure S1A). The sequencing reads with its length 5% longer than its encoded protein length covered 93% (2900/3106) of all analyzed peptides (Figure S1B). About 93% (2888/3106) proteins were inferred from at least two unique peptides (Figure S1C).

**Table 1:** **Summary of RNA-seq data from all samples**

### MMA genes of brown algae

Combined with our *Sja* data, we annotated all MMA genes in 19 brown algal species belonging to Laminariales, Ectocarpales, Desmarestiales, Dictyotales, Fucales, and Ishigeales (Table S1). Two unigenes (named *M1PDH1* and *M1PDH2* according to the naming convention of *E. siliculosus M1PDH*s) were found in most species; however, the third *M1PDH* gene (*i.e.*, *M1PDH3*) was only detected in three species belonging to Ectocarpales (Table S1)*.* The identity between *M1PDH1* and *M1PDH2* within species appeared low, ranging from 55.10% to 58.59%. The *M1Pase* genes showed a high degree of conservation in brown algae. Among all 19 species, two *M1Pase* homologs, named *M1Pase1* and *M1Pase2*, were found and they exhibited intraspecies identities from 55.61% to 68.23% (Table S2). Only one mannitol-2-dehydrogenase (*M2DH*) gene was found in 15 brown algal species (Table S3), and their identities among the species varied from 69.97% to 96.66%. Two hexokinase (*HK*) genes, *HK1* and *HK2*, were found in all 19 species, with interspecific identities of 60.98% to 67.43% (Table S4).

**Supplementary Figure 2:**
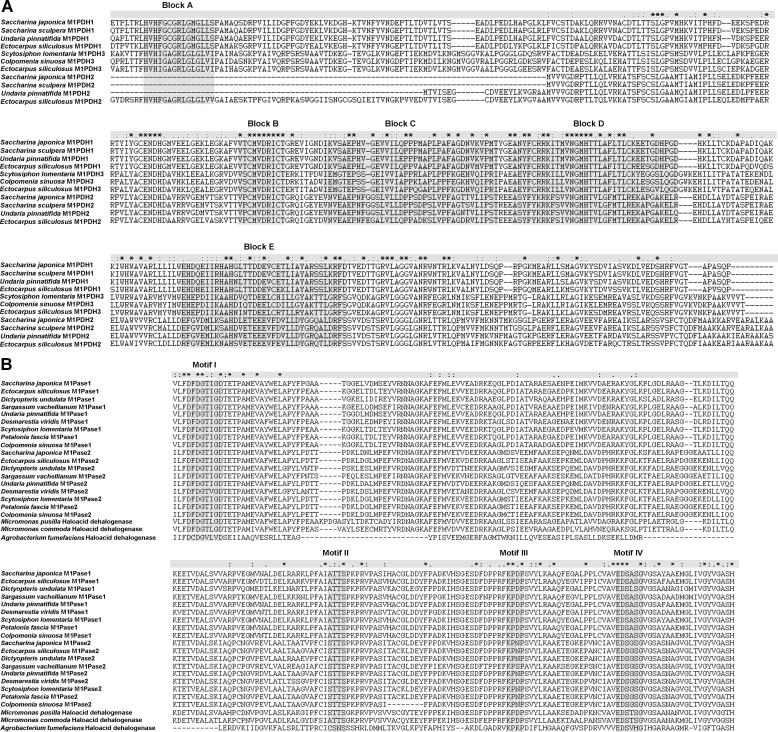
**Structure-based sequence alignment of SjaM1PDHs and SjaM1Pases A.** An amino acid sequence alignment of SjaM1PDH with its orthologs from other brown algal species. Five conserved blocks, named A to E as defined by Klimacek et al. for PSLDRs [39], are represented above the conserved consensus sequences. **B.** An amino acid sequence alignment of the crystallized HAD-like protein ATU0790 from Agrobacterium tumefaciens strain C58 (PDB code 2FDR) with M1Pase orthologs from different organisms. Motifs I–IV, defined according to Burroughs et al. [64], are represented above the conserved consensus sequences.

Having aligned the brown algal M1PDH amino acid sequences, we identified the conserved blocks A–E of the polyol-specific long-chain dehydrogenases/reductases (PSLDRs) [39]; M1PDH1 and M1PDH3 showed extended N-terminal domains compared to M1PDH2 (Figure S2A). An alignment of the brown algal M1Pase amino acid sequences with the HAD-like protein ATU0790 from *Agrobacterium tumefaciens* confirmed the shared catalytic machinery, including motifs I–IV and the Mg^2+^-cofactor-binding site (Figure S2B).

### Function confirmation of the MMA genes

We overexpressed codon-optimized *SjaM1PDH1*, *SjaM1PDH2*, *SjaM1Pase1*, and *SjaM1Pase2* in *Escherichia coli* to characterize their enzymatic activities. Although several attempts (including different vectors, expression cells, and induction conditions) were made to overexpress *SjaM1PDH1*, the efforts had failed. Then, the specificity of SjaM1PDH2 was determined by assaying its activity in the presence of different potential substrates, including F6P, G6P, and G1P for detecting reduction activity and M1P, F6P, G6P, and G1P for detecting oxidation activity. First, SjaM1PDH2 had only reduction activity in the mannitol synthesis pathway but not oxidation activity (Table 2). In addition, the reduction activity was detected for other sugar substrates (Table 3), indicating that SjaM1PDH2 is not specific for F6P. Second, purified SjaM1PDH2 had a specific activity of 0.36 μM/min/mg protein for F6P reduction with NADH at pH 8.0. This activity was in the range of those measured for algal M1PDHs, which are listed in Table 2. Using NADPH as an alternative co-factor, the F6P reduction activity of SjaM1PDH2 was less than 20% of that determined in the presence of NADH. Third, the phosphatase activity of SjaM1Pases was determined in 100 mM Tris-HCl buffer, and the specific activity of SjaM1Pase1 (144.93 μM/min/mg protein) was significantly higher (almost 22 folds) than that of SjaM1Pase2 (6.60 μM/min/mg protein) in the presence of 1 mM M1P (Table 4). Forth, the phosphatase activity of SjaM1Pases was also detected for F6P, G1P, and G6P. The activity of SjaM1Pase1 for such substrates was always lower than that for M1P, as observed for most M1Pases from brown and red algae (Table 5). However, SjaM1Pase2 exhibited the highest phosphatase activity in the presence of G1P, similar to the M1Pase from *D. grisea* showing the highest phosphatase activity using G1P (Table 5). In addition, more than 90% of the enzymatic activities were detected for SjaM1PDH2 and both SjaM1Pases after storage at 4 °C for 72 h, suggesting that the recombinant proteins are quite stable under the purification conditions.

**Table 2:** **Characterization of M1PDHs in brown and redalgae**

*Note*: NT, not tested.

**Table 3:** **M1PDH reduction activity determined in brown and redalgae**

*Note*: Percentage of maximum activities was calculated according to values reported in the different publications.

**Table 4:** **Characterization of M1Pases in brown and redalgae**

**Table 5:** **M1Pase activity determined in brown and redalgae**

*Note*: Percentage of maximum activities was calculated according to values reported in the different publications.

**Figure 2:**
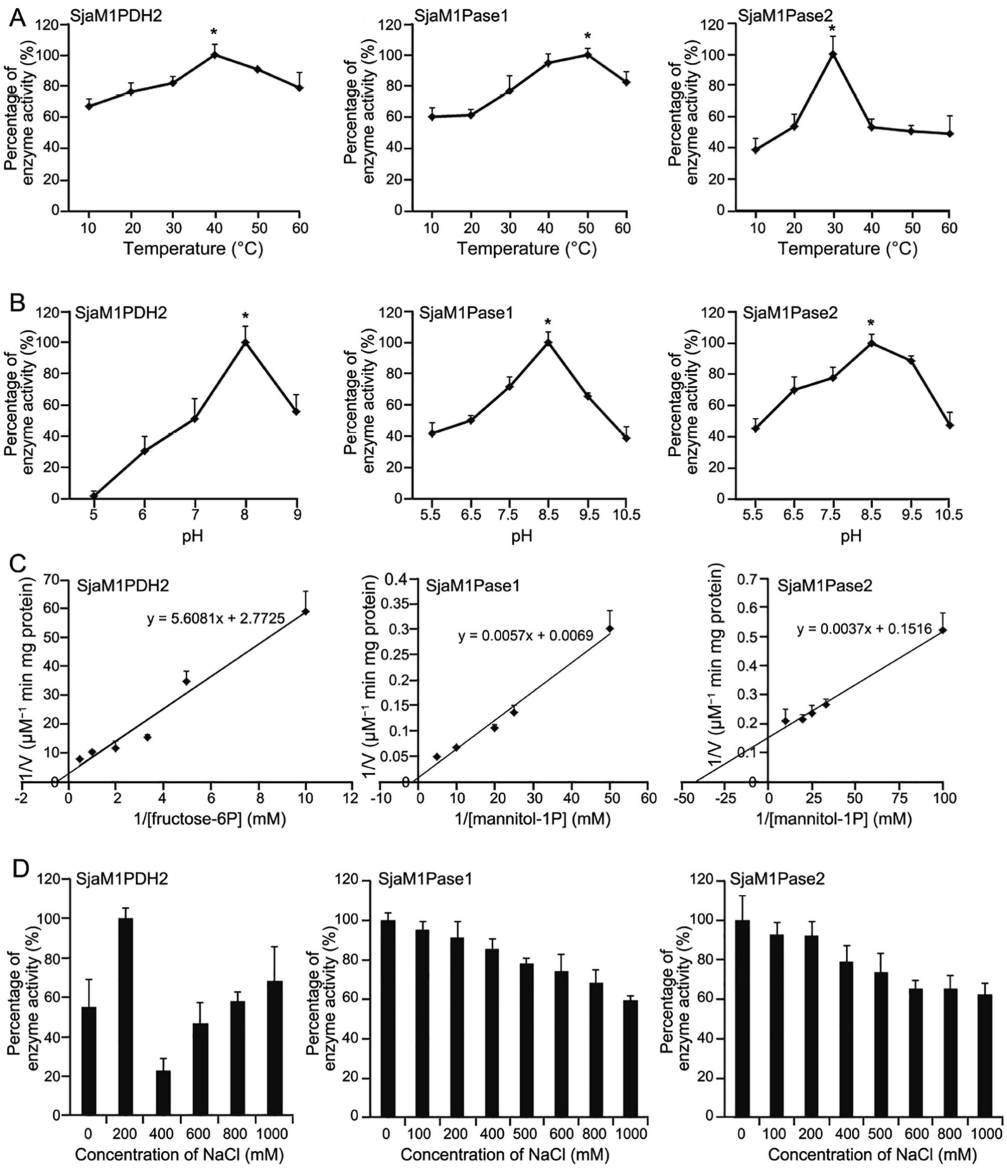
**Enzymatic characterization of recombinant SjaM1PDH2, SjaM1Pase1, andSjaM1Pase2** **A.** Temperature influence on enzyme activities of SjaM1PDH2, SjaM1Pase1, and SjaM1Pase2. The activities at 40 °C, 50 °C, and 30 °C are set to be 100% for SjaM1PDH2, SjaM1Pase1, and SjaM1Pase2, respectively. **B.** pH influence on enzyme activities of SjaM1PDH2, SjaM1Pase1, and SjaM1Pase2. The activities at pH 8.0, 8.5, and 8.5 are set to be 100% for SjaM1PDH2, SjaM1Pase1, and SjaM1Pase2, respectively. **C.** Kinetics of enzyme activities of SjaM1PDH2, SjaM1Pase1, and SjaM1Pase2. **D.** The influence of NaCl concentration on enzyme activities of SjaM1PDH2, SjaM1Pase1, and SjaM1Pase2. The values represent mean ± SD which are calculated from three replicates.

We also evaluated the enzyme activity of the three recombinants under different temperatures and pH conditions. As shown in Figure 2A, the optimal temperature for SjaM1PDH2 was 40 °C, whereas the enzyme activities were 82% and 91% of the maximum at 30 °C and 50 °C, respectively. The optimal temperature for SjaM1Pase1 was 50 °C, with 95% and 83% residual activities at 40 °C and 60 °C, respectively. The optimal temperature for SjaM1Pase2 was 30 °C, which was much lower than that of SjaM1Pase1, with less than 54% residual activities at other temperatures. As shown in Figure 2B, the optimal pH for SjaM1PDH2 was 8.0, with 51% and 56% residual activities at pH 7.0 and 9.0, respectively. The optimal pH of SjaM1Pase1 and SjaM1Pase2 was the same: 8.5.

The recombinant SjaM1PDH2 and SjaM1Pases exhibited typical Michaelis-Menten kinetics when assayed in increasing concentrations of their substrates, and the apparent *V_max_* and *K_m_* values were determined based on Lineweaver-Burk plots (Figure 2C). The *K_m_* value for SjaM1PDH2 was 2.02 mM for F6P reduction with NADH, which was approximately 10-fold higher than that for EsM1PDH1cat (0.19 mM), indicating lower substrate-binding capacity than that of EsM1PDH1cat (Table 2). The M1P substrate-binding capacities of the two SjaM1Pases were very different: the binding capacity of SjaM1Pase2 (*K_m_* = 0.02 mM) was about 41-fold higher than that of SjaM1Pase1 (*K_m_* = 0.83 mM), and it was the highest (by 21–315 folds) among the binding capacities of M1Pases from brown and red algae. Interestingly, both SjaM1Pases showed much higher catalytic efficiencies and catalytic rates than EsM1Pase2 (Table 4); SjaM1Pase1 has the highest k_cat_ value (almost four orders of magnitude higher than that of EsM1Pase2).

We then assayed the salt influence on the recombinant SjaM1PDH2 and SjaM1Pases using NaCl (Figure 2D). The results showed that SjaM1PDH2 activity altered under different NaCl conditions: it increased in the presence of 200 mM NaCl but decreased dramatically when NaCl concentration changed to 400 mM, showing a near-linear relationship from 400 mM to 1000 mM. For SjaM1Pase1 and SjaM1Pase2, a nearly linear decrease in activity was observed in the presence of NaCl at concentrations ranging from 0 mM to 1000 mM. Approximately 60% of the initial activities of both SjaM1Pases remain in the presence of 1000 mM NaCl, suggesting that these SjaM1Pases may be salt-resistant.

### Expression of the MMA genes

Our *Sja* MMA gene expression study relies on both transcriptomic and proteomic data (db.cngb.org/onekp/). All of the 19 Phaeophyceae species appear to express two *M1Pase* genes and two *HK* genes (Tables S2 and S4). Nearly 80% of the 19 Phaeophyceae species appear to express two *M1PDH* genes and one *M2DH* genes at the transcriptional level, but these genes were not identified in the remaining 20% of the Phaeophyceae species assessed in this study because of low coverage or quality of some of the transcriptomes (Tables S1 and S3). At both the transcriptomic and proteomic levels, all seven *Sja* MMA genes were detected (Tables S5 and S6), and droplet digital PCR (ddPCR) was also performed to confirm our results (Table S7).

**Figure 3:**
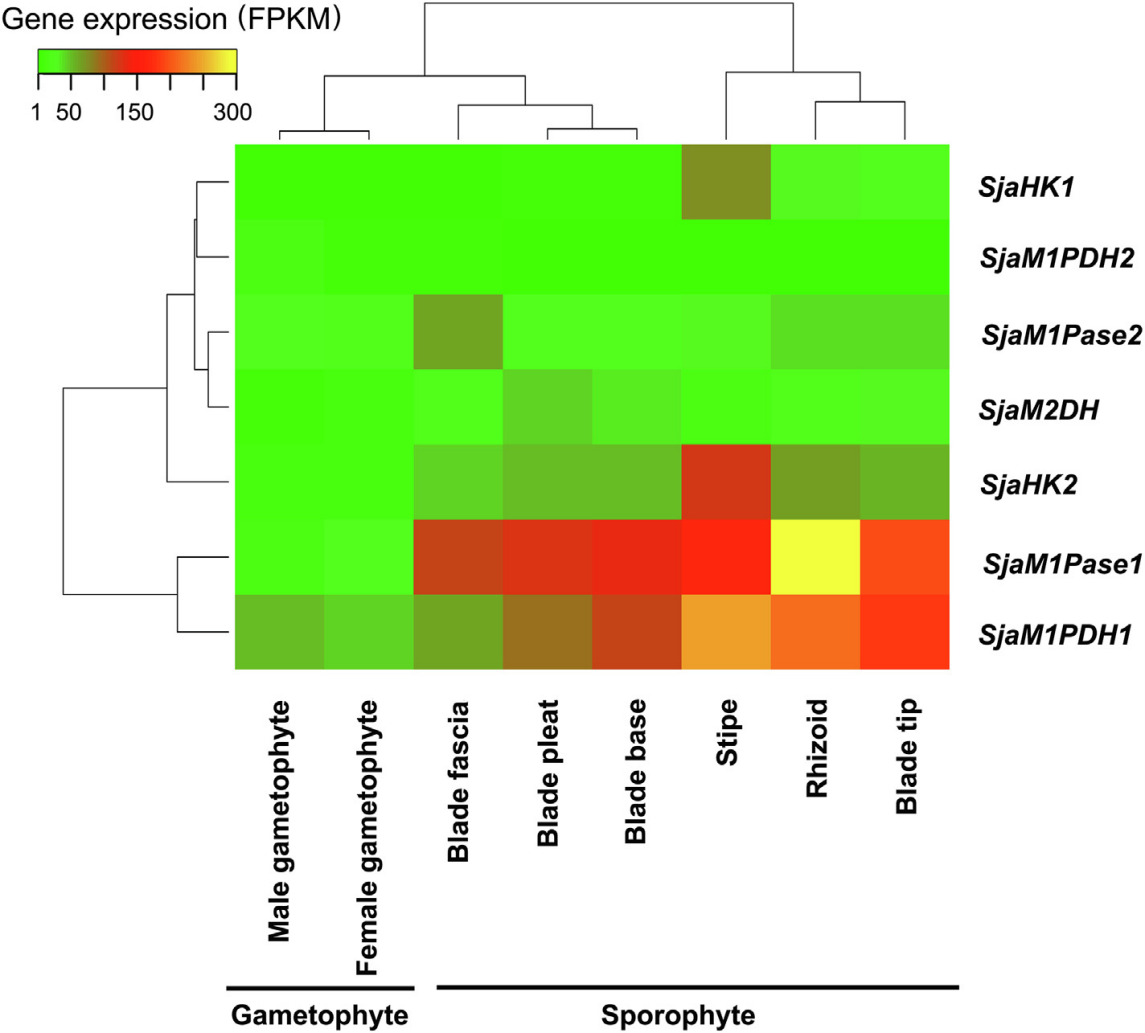
**Expression of *Sja* MMA genes in different generations andtissues** All seven *Sja* MMA genes (representing four enzyme families) were constitutively expressed in various generations (gametophytes and sporophytes) and different tissues (rhizoids, stipes, blade tips, blade pleats, blade bases, and blade fascias). The fragments per kilobase of transcript per million mapped reads (FPKM) of each gene was calculated based on the length of the gene and the number of reads mapped to the gene. The results represent mean values of three replicates.

The regulation of *Sja* mannitol metabolism is highly complex, and four observations have been made in general. First, these MMA genes (including different gene family members) were all expressed constitutively. As shown in Figure 3, all seven MMA genes (representing four enzymes) were detected in all samples. The range of fragments per kilobase of transcript per million mapped reads (FPKM) values was 1.2–300 (Table S5).

**Figure 4:**
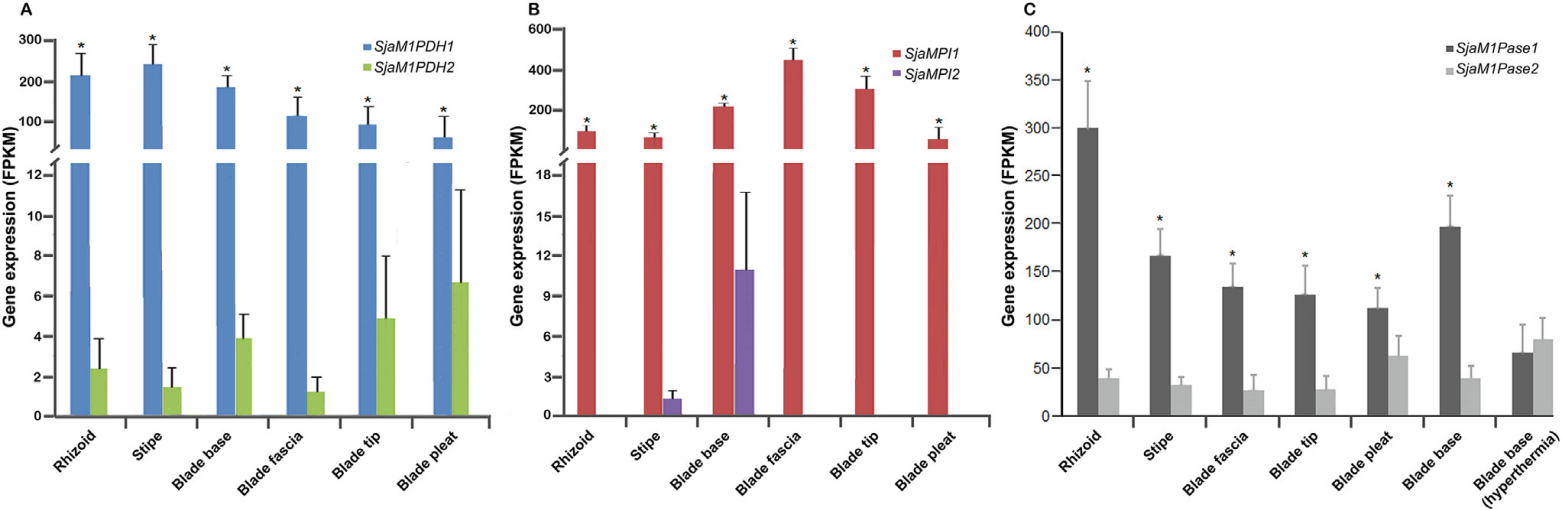
**Expression of *Sja* MMA genes in different tissues and under hyperthermiastress** **A.** The expression of *SjaM1PDH1* was significantly higher than that of *SjaM1PDH2* in all tissues. **B.** The expression of *SjaMPI1* was significantly higher than that of *SjaMPI2* in all tissues. **C.** The expression of *SjaM1Pase1* was significantly higher than that of *SjaM1Pase2* in all tissues. The expression levels of *SjaM1Pase1* and *SjaM1Pase2* were affected by hyperthermia stress (18 °C). All the data are subjected to one-way analysis of variance followed by Student’s *t*-tests. *, *P* < 0.05.

Second, most MMA genes encode reversible enzymes (except *SjaM1PDH2* and two *SjaM1Pases*), which control the balance between mannitol and F6P and dynamically maintain the “carbohydrate pool” *in vivo*. Mannitol metabolism is closely associated with the metabolism of alginate, fucoidan, laminarin, and trehalose via the intermediate product F6P. The first gene that transforms F6P to mannitol is *M1PDH* (*M1PDH1* and *M1PDH2*), and the first gene that transforms F6P to alginate and fucoidan is mannose phosphate isomerase *MPI* (*MPI1* and *MPI2*) [40]. The expression of two *SjaM1PDH* genes as well as two *SjaMPI* genes was compared among different tissues (Figure 4A and B). For example, the expression of *SjaMPI1* was 3.8-fold higher than that of *SjaM1PDH1* in blade fascias, whereas the expression of *SjaM1PDH1* is 2.7-fold higher than that of *SjaMPI1* in stipes.

Third, expression of most MMA genes differed among different tissues and under various stresses (Figures 3 and 4). Regarding to the two *M1Pase* family members, the expression of *SjaM1Pase1* was 2.0–19.4-fold higher than that of *SjaM1Pase2* in different tissues (Figure 4C). However, under hyperthermia stress (18 °C), the expression of *SjaM1Pase2* was clearly up-regulated by a 2-fold increase, whereas the expression of *SjaM1Pase1* is down-regulated by a 3-fold decrease.

**Figure 5:**
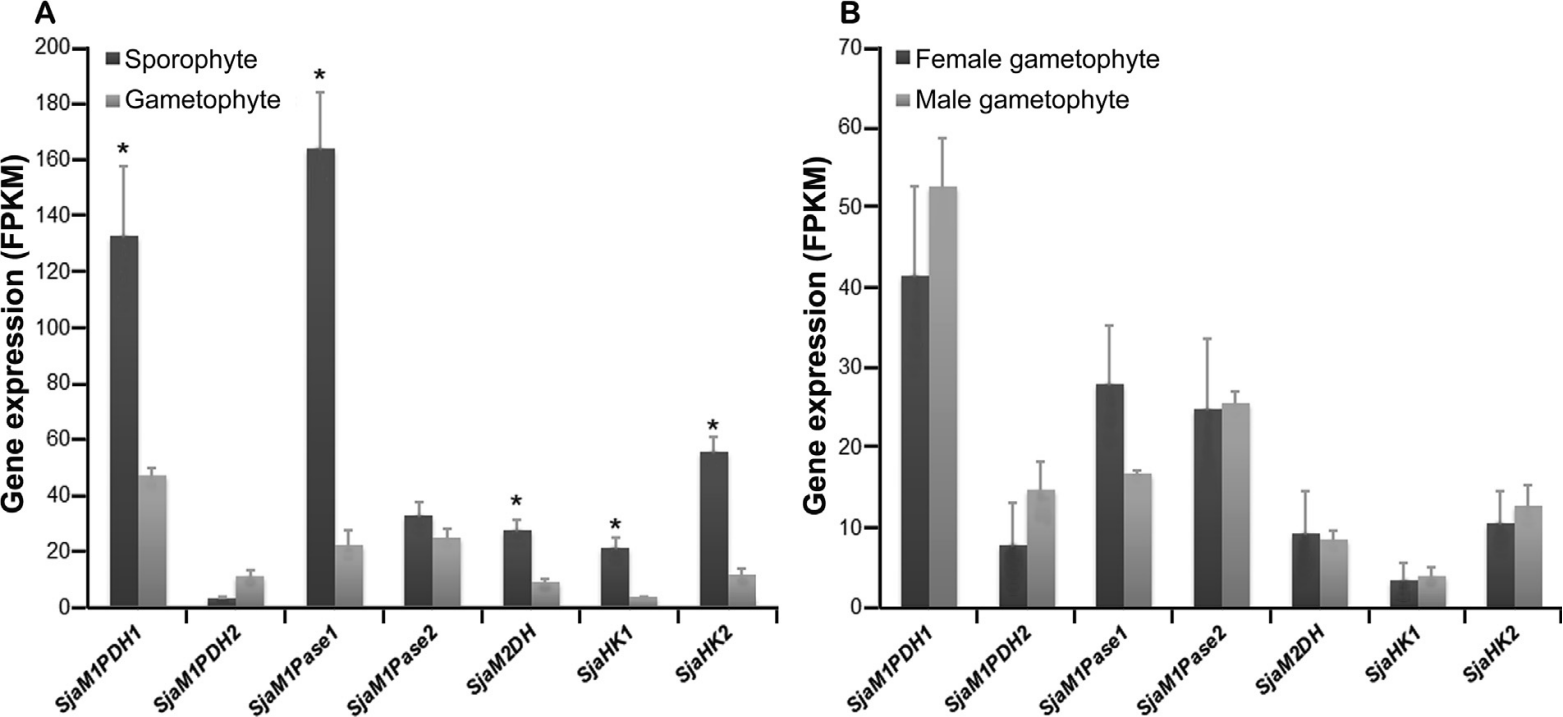
**Expression of *Sja* MMA genes of different generations andsexes** **A.** Most *Sja* MMA genes in sporophytes were expressed significantly higher than those in gametophytes. **B.** No significant expression differences were observed between sexes. All the data are subjected to one-way analysis of variance followed by Student’s t-tests. *, *P* < 0.05.

Finally, the overall gene expression profiles differed between the gametophyte and sporophyte generations. Most MMA genes (*SjaM1PDH1*, *SjaM1Pase1*, *SjaM2DH*, *SjaHK1*, and *SjaHK2*) were expressed at significantly higher levels in sporophytes than in gametophytes, showing 2.8-, 7.3-, 3.1-, 5.6-, and 4.7-fold changes, respectively (Figure 5A). However, the expression levels of *SjaM1PDH2* and *SjaM1Pase2* did not differ significantly as compared between the two generations. In addition, there was no significant expression difference among all MMA genes between the female and male gametophytes (Figure 5B).

We further investigated MMA gene expression under abiotic stresses. Under hyposaline conditions, the transcriptional levels of all MMA genes were elevated, exhibiting increases of 1.2–16.2 folds in gametophytes. In contrast, the expression levels of all MMA genes decreased in 1.9–3.7 folds in sporophytes (Table 6). Furthermore, these changes followed a similar trend under hyperthermia conditions: at the transcriptional levels, all MMA genes were up-regulated (1.2–12.5 folds) in gametophytes, whereas most of them (except *SjaM1PDH2* and *SjaM1Pase2*) were down-regulated (1.4–3.0 folds) in sporophytes (Table 6).

**Table 6:** **Ratios of FPKM values of MMA genes under differentconditions**

*Note*: Hyposaline means 12‰ NaCl and hyperthermia means 18 °C. “↑” means up-regulated fold changes and “↓” means down-regulated fold changes. *, *P* < 0.05; **, *P* < 0.01; ***, *P* < 0.001.

## Discussion

### Constitutively expressed MMA genes satisfy the requirement for mannitol biosynthesis and accumulation

Mannitol is one of the fundamental carbon-storage molecules and osmotic regulators in brown algae, and mannitol metabolism is one of the main traits that make brown algae unique as compared to other eukaryotic algae [41]. As a key metabolic pathway, mannitol metabolism has a total of four steps, and involves a limited number of gene family members (1–2 members of each gene family in most brown algae), as compared to, for instance, the *Sja* halogen metabolism (unpublished data) that has approximately 89 gene family members. As a single-product pathway, the mannitol metabolism does not contain complex synthesis- and modification-associated genes, such as those encoding glycosyltransferases, sulfurtransferases, and mannuronate C5-epimerases, as observed in alginate and fucoidan metabolism that contains dozens to more than 100 genes [40]. Therefore, mannitol metabolism is rather unique in studying pathway regulations and environmental adaptations in brown algae.

There are three *M1PDH* unigenes in *E. siliculosus*, but only two unigenes are present in the *Sja* genome. Our transcriptomic data of 19 Phaeophyceae species enable the identification of the two *M1PDH* unigenes expressed in most brown algae samples (Table S1). The presence of a third unigene of *M1PDH* in Ectocarpales may be explained by gene duplication [17]. With regard to the *M1Pase* family members, unlike other primary endosymbiotic (*e.g.*, red algae) and secondary endosymbiotic (*e.g.*, Dictyochophyceae from Ochrophyta) algae, which have only one copy of this gene [17], brown algae have two *HAD-M1Pase* genes (Table S2). In addition, our results show that all seven MMA genes are constitutively expressed in various *Sja* generations and among different *Sja* tissues (Figure 3; Table S5). In our proteomic study, proteins encoded by these MMA genes are all expressed in *Sja* female gametophytes (Table S6). Interestingly, no significant difference is observed under hyposaline and hyperthermia stresses at the proteomic level. Although up-regulation at the transcriptional level under stresses is shown as a stable trend (Table S7), the increase in gene expression doesn't necessarily imply an increased production of corresponding proteins. Even under dark conditions, all proteins involved in the pathway remain constant in *Sja* female gametophytes during diurnal cycle (Table S6).

During *Sja* development, zygotes divide continuously from a single cell to form thallus sporophytes, exhibiting consistent increases in length, width, and thickness. Mannitol of brown algae is a central compound in carbon metabolism and in the transportation and distribution of the organic assimilate. Moreover, mannitol has important physiological functions, such as osmotic regulation, thermal protection, or acting as an antioxidant and respiratory substrate [42], [43]. Therefore, these results suggest that brown algae consistently synthesize mannitol for carbon and energy storage throughout their life cycle.

### Specific MMA gene regulation in the ***Sja*** mannitol cycle

As a shared substrate, F6P can be used to synthesize not only mannitol but also other cellular components (*e.g.*, alginate and fucoidan), and MMA genes in Laminariales may be regulated in a complex integrated system. For example, the expression levels of the first genes in the mannitol and alginate/fucoidan metabolic pathways differ substantially among tissues (Figure 4A and B), indicating that abundant F6P could be utilized for the synthesis of alginate and fucoidan. This result is consistent with the finding that the accumulation of mannitol has an inverse relationship with that of alginate and fucoidan [44]. The balance between mannitol and F6P can be stringently regulated in mannitol metabolism pathway, which further affects other related pathways, such as alginate and fucoidan metabolism. It is beneficial for the maintenance of a viable carbohydrate pool *in vivo*.

### ***M1Pase1*** is a key MMA gene in mannitol synthesis

M1Pase catalyzes an irreversible reaction of the mannitol biosynthetic pathway, which is also a rate-limiting step. Although the two *M1Pase* genes appear to be expressed constitutively, they have substantial differences in expression patterns. M1Pases exhibit mannitol biosynthetic activity and have different biochemical properties. In brown algae, only one homolog of M1Pases (*i.e.*, M1Pase2) has been confirmed to be active in *E. siliculosus* [32], [38]*.* No enzymatic studies have been conducted for MIPase1; only the nucleotide sequence of *MIPase1* from *E. siliculosus* has been reported. In this study, SjaM1Pase1 and SjaM1Pase2 are both confirmed to have M1Pase activity and assumed to be involved in mannitol biosynthesis in brown algae (Figure 2). The specific enzyme activity of SjaM1Pase1 is much higher (22.0 folds) than that of SjaM1Pase2, and the former enzyme has a higher k_cat_ value (792.8 folds) than the latter (Table 4). This result indicates that SjaM1Pase1 has a higher catalytic activity than SjaM1Pase2. SjaM1Pase1 also has significantly higher expression levels than SjaM1Pase2 in all samples (Figure 4C), suggesting that *SjaM1Pase1* is the key MMA gene for mannitol production in *Sja*, whereas *SjaM1Pase2* may play complementary roles under other defined conditions. For example, while the expression of *SjaM1Pase1* is down-regulated under hyperthermia stress in sporophytes, *SjaM1Pase2* exhibits an opposite trend under the same temperature stress.

Gene duplication in evolution is an important general mechanism for maintaining normal biochemical metabolism under variable conditions, and it is probably in action here for the photosynthetic carbon storage in *Sja.* Different copies of MMA genes are observed to be transcribed, and their products exhibit activities that prevent gene loss, recombination, and mutation from affecting the pathway integrity and adequate enzymatic function. Interestingly, most genes are down-regulated under hyperthermia stress in sporophytes, whereas *SjaM1PDH2* and *SjaM1Pase2* are up-regulated (Figure 4C, Table S5). These findings suggest that paralogs encoding proteins with the same enzyme activity may be regulated by different mechanisms and/or under difference environmental conditions. In addition, SjaM1PDH2 lacks M1P oxidation activity. A previous study has shown that EsM1PDH1cat can oxidize M1P [38]. In our analysis, SjaM1PDH1 and EsM1PDH1cat have highly conserved domains (Blocks A–E, Figure S2), and we thus speculate that the M1P oxidation activity may be conducted by SjaM1PDH1 in *Sja*, whereas SjaM1PDH2 only maintains the reduction activity. However, the reduction activity of SjaM1PDH2 is not specific to F6P, suggesting that it may be involved in other metabolic pathway(s), such as phosphoglucose reduction. This functional differentiation between homologous genes is similar to the *MPI* genes in alginate/fucoidan metabolism [40].

### The complex regulation of MMA genes is beneficial for brown algal evolution and adaptation

Laminariales have a heteromorphic haploid-diploid life cycle, with a macroscopic thallus sporophyte and a microscopic gametophyte among different generations [19]. Most MMA genes (except *SjaM1PDH2* and *SjaM1Pase2*) exhibit significantly higher expression in sporophytes (thallus generation) than in gametophytes (filamentous generation) (Figure 5A). These results indicate that sporophytes may have a much greater ability to synthesize mannitol than gametophytes, potentially because sporophytes (large thallus with tissue differentiation) require high levels of mannitol not only for normal growth and development, but also for adaptation to changing environments. However, there are no significant differences in gene expression between female and male gametophytes (Figure 5B). This result is consistent with our observation that the mannitol contents in male and female gametophytes are very similar in quantity, 23.4% and 24.6% (*P* > 0.05), respectively (unpublished data).

The expression levels of MMA genes in response to environmental stresses show opposite trends between gametophyte and sporophyte generations. Under hyposaline stress, the expression of all MMA genes in gametophytes is up-regulated, consistent with the results observed for *M1Pase2* in *E. siliculosus* [32]; on the contrary, the expression of all MMA genes in sporophytes is down-regulated (Table 6). Similar results are observed under hyperthermia condition. Notably, mannitol is the main organic osmolyte in *Sja* and most other brown algae, counteracting salinity stress and acting as an antioxidant and heat protectant for protein stabilization [1], [42], [43]. The gametophyte stage is most vulnerable to external stresses in the entire life cycle [45]. Therefore, in this stage, mannitol metabolism is increased in order to respond to stresses, particularly hyposaline. Distinct regions of carbon sources and carbon sinks exist along thalli because of their large size and morphological differentiation [46], [47]. *Saccharina* species are capable of transporting carbons from source (mature blade areas) to sink, producing a surplus of photoassimilates transported to intercalary carbon-requiring meristems [19]. The imported organic compounds in the sink tissues are rapidly metabolized and incorporated into polysaccharides and proteins [46]. The blade bases of the sporophytes (meristem) exhibit reduced mannitol catabolism, indicating a reduction of mannitol degradation and incorporation [48]. We speculate that this condition may decrease transportation from carbon sources to carbon sinks and increase mannitol accumulation in mature blades for stress responses.

### The mutual validation of RNA-seq and ddPCR results provides confidence in copy number quantitation

We performed ddPCR, a microfluidics-based technique for determination of DNA or RNA copy number in a given sample, to verify our key RNA-seq results in female gametophytes under stress conditions (hyposaline and hyperthermia). Most of our gene expression results are confirmed. For example, in RNA-seq analysis, *SjaM1PDH1* expression shows 3.75- and 1.87-fold increases under hyposaline and hyperthermia stresses, respectively, and the ddPCR analysis accordingly shows 3.97- and 1.30-fold increases, respectively (Table S7). Notably, ddPCR is relatively inexpensive and has shorter experimental turnaround time of RNA-seq.

### Characterization of MMA genes is essential for future industrial production and genetic breeding

Mannitol is a commercially valuable compound widely used in food, pharmaceutical, medical, and chemical industries [23], [49]. Most of mannitol commercial production is carried out by chemical hydrogenation of fructose or by extraction from seaweed [13], [23]. Since environmental issues associate with chemical refine and production, microbe-based alternatives have been a subject of significant interest in recent years [23]. The most widely used *M1Pase* gene, from the protozoan parasite *Eimeria tenella* [31], has been expressed in Cyanobacteria [50], Proteobacteria [51], and Firmicutes [52] to generate cellular or extracellular mannitol. Interestingly, the substrate-binding capacity of the *E. tenella* M1Pase (*K_m_* = 0.07 mM) is lower than that of SjaM1Pase2 (*K_m_* = 0.02 mM), and the catalytic efficiency of the *E. tenella* M1Pase (k_cat_ = 430 s^−1^) is much lower than that of SjaM1Pase1 (k_cat_ = 6453.48 s^−1^), indicating that the *M1Pase* genes from brown algae may be suitable candidates for engineered microbes for mannitol production. Furthermore, introduction of algal genes into transgenic plants may confer great advantage in terms of salt tolerance [33]. For example, mannitol biosynthesis by genetic engineering is one of the most extensively tested alternatives for improving salinity tolerance in plants [1]. The high salinity tolerance of transgenic plants may be a result of both the accumulation of mannitol in cells [53], [54] and increased expression of a variety of stress-inducible genes [55]. Analysis of MMA genes in the mannitol synthesis pathway helps identify enzymes with high substrate specificities and activities, which are useful for genetic breeding of both algae and plants in the future.

## Materials and methods

### Algal sample collection

Preserved *Sja* haploid gametophytes (male and female gametophytes) were available in our laboratory cultures (Laboratory of Genetics and Breeding of Marine Organisms, Ocean University of China), which were grown in a modified seawater medium supplemented with nutrients (4 mg/L NaNO_3_-N and 0.4 mg/L KH_2_PO_4_-P) at 10 °C under 30 μM photons m^−2^ s^−1^ irradiance. Fresh samples of *Sja* sporophytes were collected from eastern China (Rongcheng, Shandong Province, 37°8′53″N, 122°34′33″E) and kept in the laboratory under the same culture conditions as gametophytes for a few days to acclimate, and then dissected to obtain different tissues (rhizoids, stipes, blade tips, blade pleats, blade bases, and blade fascias). To study the effects of abiotic factors, the female gametophytes (5 g each sample) and blade bases of sporophytes (5 g each sample) were cultured at different temperatures (control: 8 °C; hyperthermia: 18 °C) and salinities (control: 30‰; hyposaline: 12‰) for 6 h; the female gametophytes (5 g each sample) were cultured under a 12-h/12-h photoperiod (control: collected at the end of 12-h light period; darkness: collected at the end of 12-h dark period).

### Sequence analysis

Based on the analysis of the *Sja* genome sequence downloaded from Genome Warehouse (GWH: GWHAAET00000000; http://bigd.big.ac.cn/gwh/browse/index), the transcriptome database downloaded from 1000 plant trancriptomes (OneKP: OGZM; https://db.cngb.org/onekp/), and the transcriptome and proteome databases obtained in our study, the unigenes related to mannitol metabolism pathway were verified using the BLASTX algorithm (http://blast.ncbi.nlm.nih.gov/Blast.cgi). Multiple sequence alignments were performed with ClustalX [56]. Sequence identities were calculated using the Clustal Omega tool (http://www.ebi.ac.uk/Tools/msa/clustalo/).

### Purification of recombinant proteins expressed in ***E. coli***

Genes were codon-optimized and synthesized (Shanghai Xuguan Biotechnological Development Co., Ltd, Shanghai, China) to construct recombinant plasmids. *SjaM1PDH1*, *SjaM1PDH2*, and *SjaM1Pase1* were cloned into pET32a, and *SjaM1Pase2* was cloned into pGEX-6p-1. The inserts were cloned into the plasmids between the *Eco*RI and *Not*I sites. After ligation, the generated recombinant plasmids were transformed into *E. coli* BL21 (DE3) cells, and the integrity of the sequences was verified based on Sanger sequencing.

The recombinant strains were cultivated in 1 L LB liquid medium. When the optical density at 600 nm (OD_600_) reached 0.6, isopropyl β-D-1-thiogalactopyranoside (IPTG) was added at a final concentration of 0.5 mM to induce overexpression of the target genes to produce recombinant proteins, and the bacterial cultures were incubated for 16 h at 20 °C. Then, the cells were harvested by centrifugation at 12,000 *g* for 3 min at 4 °C, suspended in 20 ml of 50 mM Tris-HCl buffer (pH 8.0), and lysed by sonication (Selecta Sonopuls, Shanghai, China) to release the recombinant proteins. His-binding resin and GST-binding resin were used to purify the recombinant proteins according to the manufacturer’s instructions (www.yuekebio.com). The proteins were stored at −80 °C.

### Enzyme kinetic assays

The enzyme activities of recombinant SjaM1PDH2, SjaM1Pase1, and SjaM1Pase2 were determined using previously described methods [32], [38]. For enzymatic characterization, four sugar and polyol phosphoesters considered as potential substrates were tested: M1P, F6P, G1P, and G6P (Sigma, St. Louis, MO). The pH effect on the enzyme activity was determined at pH values ranging from 5.0 to 9.0 for SjaM1PDH2 and 5.5 to 10.5 for SjaM1Pases. The temperature effect was determined at temperatures ranging from 10 °C to 60 °C. The NaCl influence was assessed at final concentrations ranging from 0 mM to 1000 mM in the reaction mixture. Four replicates were analyzed for each condition to ensure reproducibility of the experimental results. In each case, the heat-denatured recombinant enzyme was used as a negative control.

### RNA-seq and ddPCR

Total RNA was extracted by using an improved CTAB method [57]. Three micrograms of total RNA per sample were used for the construction of sequencing libraries. mRNAs were enriched by using oligo-dT beads, and sequencing libraries were generated by using the NEBNext Ultra RNA Library Prep Kit (NEB, Ipswich, MA) for Illumina according to the manufacturer’s instructions. Then, index codes were added to attribute sequences to each sample. Clustering of the index-coded samples was performed on a cBot cluster generation system (TruSeq PE Cluster Kit v3-cBot-HS, Illumina, San Diego, CA) according to the manufacturer’s instructions. After cluster generation, the libraries were sequenced on the Illumina HiSeq platform, and the length of paired-end reads was 2 × 150 bp. High-quality reads (quality above Q20 sequences, no adaptor contamination, and no ambiguous N bases) were aligned to the *Sja* genome (NCBI accession No. JXRI00000000.1) and to a set of gene model annotations including MMA genes using HISAT2 [58]. HTSeq v0.6.1 was used to count the read numbers mapped to each gene [59]. The FPKM of each gene was then calculated based on the length of the gene and the number of reads mapped to the gene.

ddPCR analysis was conducted according to previously described methods [40]. Each 20 μl reaction mixture contained: 1× Droplet PCR Supermix (Bio-Rad, Hercules, CA), 3 μl of sample cDNA, 900 nM of each primer, and 250 nM of the probe (Table S8). The 20 μl mixture was mixed with 70 μl droplet generation oil in the Droplet Generator (Bio-Rad, Hercules, CA) via microfluidics. The water-in-oil droplets were transferred to a standard 96-well PCR plate for PCR amplification. The PCR program included an initial denaturing step at 95 °C for 10 min, followed by 40 cycles of 94 °C for 30 s and 60 °C for 60 s, and a final step at 98 °C for 10 min. The results represented mean values of three replicates. All data were subjected to one-way analysis of variance followed by Student’s *t*-test.

### Protein identification by LC–MS/MS

Every five samples, including three treatments, a control, and an internal standard (IS; all samples pooled equally), were labeled as a replicate, and three replicates were taken for each measurement. Proteins were extracted using the phenol/SDS method described by Nagai K et al. [60]. Protein digestion was conducted according to the filter-aided sample preparation (FASP) procedure [61]. TMT6plex labeling was conducted according to the manufacturer’s recommendations (ThermoFisher Scietific, Waltham, MA). The TMT-labeled peptides were subjected to high-pH reversed-phase fractionation in an 1100 Series HPLC Value system (Agilent, Santa Clara, CA) equipped with a Gemini-NX (00F-4453-E0, Phenomenex, Torrance, CA) column (4.6 mm × 150 mm, 3 µm, 100 Å). The collected 45 fractions were concentrated via vacuum centrifugation and reconstituted in 40 µl of 0.1% v/v trifluoroacetic acid. Then, the TMT-labeled samples were analyzed using an Easy-nLC nanoflow HPLC system (ThermoFisher Scietific), which was connected to an Orbitrap Fusion mass spectrometer (ThermoFisher Scietific).

The raw data were analyzed using Proteome Discoverer 2.1 software (ThermoFisher Scietific) with mascot 2.3 against the protein database translated from the *Saccharina japonica* transcriptome (GSA: PRJCA000815). The search parameters were used as described by Chen L et al. [62]. The final ratios obtained from the relative protein quantifications were normalized based on the IS and median average protein quantification ratio followed by Student's *t*-test for *P* value.

## Data availability

The raw sequence data are deposited in the Genome Sequence Archive [63] at the National Genomics Data Center, Beijing Institute of Genomics, Chinese Academy of Sciences / China National Center for Bioinformation (GSA: PRJCA000815), and are publicly accessible at http://bigd.big.ac.cn/gsa. The raw protein data and metadata are deposited in  the ProteomeXchange Consortium via the iProX partner repository (ProteomeXchange: PXD009642), and are publicly accessible at http://proteomecentral.proteomexchange.org. The cDNA sequences of *SjaM1PDH* and *SjaM1Pase* genes are deposited in GenBank: *SjaM1PDH1* (GenBank: MF706368), *SjaM1PDH2* (GenBank: MF706369), *SjaM1Pase1* (GenBank: MF440344), and *SjaM1Pase1* (GenBank: MF465902).

## CRediT author statement

**Shan Chi:** Methodology, Formal analysis, Visualization, Writing - original draft, Writing - review & editing. **Guoliang Wang:** Methodology, Formal analysis, Data curation, Visualization, Software, Writing - original draft, Writing - review & editing. **Tao Liu:** Conceptualization, Resources, Supervision, Funding acquisition. **Xumin Wang:** Conceptualization, Resources, Supervision. **Cui Liu:** Investigation. **Yuemei Jin:** Investigation. **Hongxin Yin:** Investigation. **Xin Xu:** Investigation. **Jun Yu:** Conceptualization, Resources, Writing - review & editing, Supervision. All authors read and approved the final manuscript.

## Competing interests

The authors have declared no competing interests.

## Supplementary material

Supplementary Table S1 ***M1PDH* genes identified in 19 Phaeophyceae****species**

Supplementary Table S2 ***M1Pase* genes identified in 19 Phaeophyceae****species**

Supplementary Table S3 ***M2DH* genes identified in 19 Phaeophyceae****species**

Supplementary Table S4 ***HK* genes identified in 19 Phaeophyceae****species**

Supplementary Table S5 **FPKM values of *Sja* MMA****transcripts**

Supplementary Table S6 **The individual protein spectral counts of *Sja* gametophytes based on TMT quantitative proteomics****analysis**

Supplementary Table S7 **The upregulated ratios of *Sja* MMA transcripts under stress conditions based on ddPCR and RNA-seq****analyses**

Supplementary Table S8 **Primers and probes used in droplet digital****PCR**
