# Supplementary material for: Transcriptomic and Proteomic Analysis of Mannitol-metabolism-associated Genes in Saccharina japonica
Source: Genomics Proteomics Bioinformatics. 2020 Nov 25;18(4):415–29. doi: 10.1016/j.gpb.2018.12.012 (PMC8242268; doi:10.1016/j.gpb.2018.12.012)
Supplement: Supplementary Table S1 — M1PDH genes identified in 19 Phaeophyceaespecies [file mmc1.docx]

**Table S1 *M1PDH* genes identified in 19 Phaeophyceae species**

| Species | *M1PDH1* (bp) | *M1PDH2* (bp) | Identity between *M1PDH1* and *M1PDH2* (%) | *M1PDH3* (bp) |
| --- | --- | --- | --- | --- |
| *Saccharina japonica* | MF706368 (1698) | MF706369 (1068) | 58.48 | - |
| *Colpomenia sinuosa* | QLMZ-2012163 (1674) | QLMZ-2003277 (1914) | 56.65 | QLMZ-2011570 (1692) |
| *Desmarestia viridis* | FSQE-2004782 (1677) | FSQE-2051959 (1017) | 56.58 | - |
| *Dictyopteris undulata* | LIRF-2100046 (1752) | LIRF-2099681 (1671) | 55.30 | - |
| *Ectocarpus siliculosus* | Esi0017_0062 (1578) | Esi0080_0017 (1293) | 56.98 | Esi0020_0181 (1662) |
| *Ishige okamurai* | APTP-2013531 (1587) | APTP-2078175 (1962) | 55.10 | - |
| *Petalonia fascia* | VRGZ-2089225 (1680) | - | - | - |
| *Punctaria latifolia* | - | ASZK-2021123 (978) | - | - |
| *Saccharina sculpera* | RAPY-2007233 (1698) | RAPY-2086441 (1068) | 58.59 | - |
| *Sargassum hemiphyllum* var.chinense | VYER-2012569 (1680) | VYER-2086764 (1047) | 56.10 | - |
| *Sargassum henslowianum* | FIKG-2011971 (1680) | FIKG-2003445 (1047) | 55.35 | - |
| *Sargassum horneri* | RWXW-2013594 (1680) | RWXW-2016232 (1047) | 55.58 | - |
| *Sargassum integerrimum* | FOMH-2013956 (1584) | FOMH-2016311 (1047) | 55.53 | - |
| *Sargassum muticum* | JGGD-2003565 (1680) | JGGD-2015649 (1047) | 57.37 | - |
| *Sargassum thunbergii* | YRMA-2000913 (1584) | - | - | - |
| *Sargassum vachellianum* | HFIK-2002026 (1584) | HFIK-2069662 (1653) | 56.25 | - |
| *Scytosiphon lomentaria* | JCXF-2075599 (1677) | JCXF-2008474 (1884) | 55.44 | JCXF-2011379 (1671) |
| *Scytosiphon dotyi* | ULXR-2015500 (1680) | - | - | - |
| *Undaria pinnatifida* | FIDQ-2013542 (1581) | FIDQ-2071234 (1122) | 57.75 | - |
