# Supplementary material for: Transcriptomic and Proteomic Analysis of Mannitol-metabolism-associated Genes in Saccharina japonica
Source: Genomics Proteomics Bioinformatics. 2020 Nov 25;18(4):415–29. doi: 10.1016/j.gpb.2018.12.012 (PMC8242268; doi:10.1016/j.gpb.2018.12.012)
Supplement: Supplementary Table S2 — M1Pase genes identified in 19 Phaeophyceaespecies [file mmc2.docx]

**Table S2 *M1Pase* genes identified in 19 Phaeophyceae species**

| Species | *M1Pase1* (bp) | *M1Pase2* (bp) | Identity (%) |
| --- | --- | --- | --- |
| *Saccharina japonica* | MF440344 (1209) | MF465902 (984) | 63.48 |
| *Colpomenia sinuosa* | QLMZ-2008139 (1245) | QLMZ-2016866 (984) | 61.82 |
| *Desmarestia viridis* | FSQE-2003898 (1212) | FSQE-2051711 (984) | 65.35 |
| *Dictyopteris undulata* | LIRF-2099575 (1230) | LIRF-2010393 (987) | 61.36 |
| *Ectocarpus siliculosus* | Esi0080_0016 (1218) | Esi0100_0020 (984) | 67.14 |
| *Ishige okamurai* | APTP-2012816 (1134) | APTP-2011070 (987) | 55.61 |
| *Petalonia fascia* | VRGZ-2087949 (1230) | VRGZ-2087851 (990) | 67.68 |
| *Punctaria latifolia* | ASZK-2098041 (1237) | ASZK-2017609 (960) | 66.63 |
| *Saccharina sculpera* | RAPY-2011639 (1209) | RAPY-2011301 (984) | 67.51 |
| *Sargassum hemiphyllum* var.chinense | VYER-2086141 (1272) | VYER-2086071 (987) | 57.32 |
| *Sargassum henslowianum* | FIKG-2076836 (1272) | FIKG-2008421 (987) | 63.45 |
| *Sargassum horneri* | RWXW-2074720 (1272) | RWXW-2009468 (987) | 57.70 |
| *Sargassum integerrimum* | FOMH-2082257 (1272) | FOMH-2009194 (987) | 57.60 |
| *Sargassum muticum* | JGGD-2080538 (1272) | JGGD-2000240 (987) | 57.54 |
| *Sargassum thunbergii* | YRMA-2105805 (1272) | YRMA-2002970 (987) | 63.80 |
| *Sargassum vachellianum* | HFIK-2068508 (1272) | HFIK-2009162 (987) | 58.13 |
| *Scytosiphon lomentaria* | JCXF-2007460 (1257) | JCXF-2013549 (990) | 67.79 |
| *Scytosiphon dotyi* | ULXR-2069055 (1248) | ULXR-2014755 (990) | 68.23 |
| *Undaria pinnatifida* | FIDQ-2071780 (1227) | FIDQ-2007939 (984) | 59.77 |
