# Supplementary material for: Transcriptomic and Proteomic Analysis of Mannitol-metabolism-associated Genes in Saccharina japonica
Source: Genomics Proteomics Bioinformatics. 2020 Nov 25;18(4):415–29. doi: 10.1016/j.gpb.2018.12.012 (PMC8242268; doi:10.1016/j.gpb.2018.12.012)
Supplement: Supplementary Table S3 — M2DH genes identified in 19 Phaeophyceaespecies [file mmc3.docx]

**Table S3 *M2DH* genes identified in 19 Phaeophyceae species**

| Species | *M2DH* (bp) |
| --- | --- |
| *Saccharina japonica* | SRSQ-2004646(2007) |
| *Colpomenia sinuosa* | QLMZ-2009651 (1998) |
| *Desmarestia viridis* | FSQE-2008418 (1986) |
| *Dictyopteris undulata* | - |
| *Ectocarpus siliculosus* | Esi0135_0010 (2001) |
| *Ishige okamurai* | APTP-2012200 (2031) |
| *Petalonia fascia* | - |
| *Punctaria latifolia* | ASZK-2022510 (1992) |
| *Saccharina sculpera* | RAPY-2087783 (2004) |
| *Sargassum hemiphyllum* var.chinense | VYER-2016703 (2007) |
| *Sargassum henslowianum* | FIKG-2015971 (2007) |
| *Sargassum horneri* | - |
| *Sargassum integerrimum* | FOMH-2002399 (2013) |
| *Sargassum muticum* | JGGD-2014526 (2010) |
| *Sargassum thunbergii* | YRMA-2106092 (2007) |
| *Sargassum vachellianum* | HFIK-2012284 (2007) |
| *Scytosiphon lomentaria* | JCXF-2011980 (1998) |
| *Scytosiphon dotyi* | ULXR-2069338 (1998) |
| *Undaria pinnatifida* | *-* |
