# Supplementary material for: Transcriptomic and Proteomic Analysis of Mannitol-metabolism-associated Genes in Saccharina japonica
Source: Genomics Proteomics Bioinformatics. 2020 Nov 25;18(4):415–29. doi: 10.1016/j.gpb.2018.12.012 (PMC8242268; doi:10.1016/j.gpb.2018.12.012)
Supplement: Supplementary Table S4 — HK genes identified in 19 Phaeophyceaespecies [file mmc4.docx]

**Table S4 *HK* genes identified in 19 Phaeophyceae species**

| Species | *HK1* (bp) | *HK2* (bp) | Identity (%) |
| --- | --- | --- | --- |
| *Saccharina japonica* | SRSQ-2095653(840)-partial | SRSQ-2018380(1506) | - |
| *Colpomenia sinuosa* | QLMZ-2017730 (1959) | QLMZ-2009579 (1467) | 63.58 |
| *Desmarestia viridis* | FSQE-2006825 (1632) | FSQE-2008483 (1506) | 67.43 |
| *Dictyopteris undulata* | LIRF-2016209 (1899) | LIRF-2100131 (1500) | 65.17 |
| *Ectocarpus siliculosus* | Esi0437_0013 (1650) | Esi0216_0030 (1467) | 60.98 |
| *Ishige okamurai* | APTP-2000408 (1929) | APTP-2008740 (1488) | 63.69 |
| *Petalonia fascia* | VRGZ-2012430 (1959) | VRGZ-2017436 (1443) | 64.78 |
| *Punctaria latifolia* | ASZK-2012271 (1632) | ASZK-2017344 (1443) | 67.25 |
| *Saccharina sculpera* | RAPY-2014084 (1932) | RAPY-2016616 (1501)-partial | - |
| *Sargassum hemiphyllum* var.chinense | VYER-2086637 (1638) | VYER-2086836 (1533) | 63.51 |
| *Sargassum henslowianum* | FIKG-2077221 (1638) | FIKG-2013984 (1527) | 63.27 |
| *Sargassum horneri* | RWXW-2015824 (1656) | RWXW-2013629 (1521) | 62.67 |
| *Sargassum integerrimum* | FOMH-2082404 (1638) | FOMH-2017786 (1527) | 63.69 |
| *Sargassum muticum* | JGGD-2003186(1614)-partial | JGGD-2016599 (1521) | - |
| *Sargassum thunbergii* | YRMA-2106203 (1638) | YRMA-2106406 (1530) | 63.09 |
| *Sargassum vachellianum* | HFIK-2010153 (1374)-partial | HFIK-2010038 (1513)-partial | - |
| *Scytosiphon lomentaria* | JCXF-2075382 (1965) | JCXF-2074998 (1461) | 66.48 |
| *Scytosiphon dotyi* | ULXR-2010142 (1944) | ULXR-2018006 (1461) | 65.70 |
| *Undaria pinnatifida* | FIDQ-2013200 (1461)-partial | FIDQ-2072068 (1503) | - |
