# Supplementary material for: Transcriptomic and Proteomic Analysis of Mannitol-metabolism-associated Genes in Saccharina japonica
Source: Genomics Proteomics Bioinformatics. 2020 Nov 25;18(4):415–29. doi: 10.1016/j.gpb.2018.12.012 (PMC8242268; doi:10.1016/j.gpb.2018.12.012)
Supplement: Supplementary Table S5 — FPKM values of Sja MMAtranscripts [file mmc5.docx]

**Table S5 FPKM values of *Sja* MMA transcripts**

| **Samples** | ***SjaM1PDH*** | |  | ***SjaM1Pase*** | |  | ***SjaM2DH*** |  | ***SjaHK*** | |
| --- | --- | --- | --- | --- | --- | --- | --- | --- | --- | --- |
|  | ***SjaM1PDH1*** | ***SjaM1PDH2*** |  | ***SjaM1Pase1*** | ***SjaM1Pase2*** |  |  |  | ***SjaHK1*** | ***SjaHK2*** |
| Sporophytes |  |  |  |  |  |  |  |  |  |  |
| Rhizoids | 213.71 | 2.32 |  | 300.08 | 39.56 |  | 23.31 |  | 31.26 | 65.27 |
| Stipe | 240.85 | 1.42 |  | 166.86 | 31.98 |  | 15.75 |  | 75.82 | 122.65 |
| Blade tip | 92.03 | 4.81 |  | 126.41 | 27.56 |  | 42.07 |  | 7.39 | 52.20 |
| Blade pleat | 61.57 | 6.61 |  | 112.93 | 62.51 |  | 23.49 |  | 3.20 | 41.16 |
| Blade fascia | 113.74 | 1.21 |  | 134.50 | 26.95 |  | 36.83 |  | 8.08 | 49.59 |
| Blade base | 183.91 | 3.84 |  | 196.37 | 38.98 |  | 32.27 |  | 29.02 | 56.65 |
| Blade base (hyposaline) | 62.42 | 1.04 |  | 104.39 | 11.23 |  | 11.97 |  | 8.13 | 16.20 |
| Blade base (hyperthermia) | 93.70 | 21.38 |  | 66.17 | 79.73 |  | 18.59 |  | 9.97 | 40.58 |
| Gametophytes |  |  |  |  |  |  |  |  |  |  |
| Male gametophyte | 52.64 | 14.79 |  | 16.79 | 25.69 |  | 8.62 |  | 4.07 | 12.85 |
| Female gametophyte | 41.46 | 7.83 |  | 27.98 | 24.83 |  | 9.36 |  | 3.42 | 10.65 |
| Female gametophyte (hyposaline) | 155.37 | 9.42 |  | 53.53 | 49.62 |  | 151.52 |  | 15.85 | 70.31 |
| Female gametophytes (hyperthermia) | 77.43 | 61.51 |  | 76.66 | 29.87 |  | 117.03 |  | 4.56 | 24.82 |
