# Supplementary material for: Transcriptomic and Proteomic Analysis of Mannitol-metabolism-associated Genes in Saccharina japonica
Source: Genomics Proteomics Bioinformatics. 2020 Nov 25;18(4):415–29. doi: 10.1016/j.gpb.2018.12.012 (PMC8242268; doi:10.1016/j.gpb.2018.12.012)
Supplement: Supplementary Table S6 — The individual protein spectral counts of Sja gametophytes based on TMT quantitative proteomicsanalysis [file mmc6.docx]

**Table S6 The individual protein spectral counts of *Sja* gametophytes based on TMT quantitative proteomics analysis**

| **Protein** | **Female gametophyte** | **Female gametophyte (hyposaline)** | **Female gametophyte (hyperthermia)** | **Female gametophyte (darkness)** |
| --- | --- | --- | --- | --- |
| SjaM1PDH1 | 1.08 | 1.01 | 0.99 | 0.99 |
| SjaM1PDH2 | 1.02 | 1.00 | 0.98 | 0.98 |
| SjaM1Pase1 | 1.30 | 1.74 | 1.48 | 1.47 |
| SjaM1Pase2 | 0.99 | 1.01 | 1.00 | 1.00 |
| SjaM2DH | 0.90 | 1.02 | 1.02 | 1.00 |
| SjHK1 | 1.03 | 0.98 | 0.99 | 1.00 |
| SjHK2 | 1.02 | 0.97 | 0.97 | 0.95 |
