# Supplementary material for: Transcriptomic and Proteomic Analysis of Mannitol-metabolism-associated Genes in Saccharina japonica
Source: Genomics Proteomics Bioinformatics. 2020 Nov 25;18(4):415–29. doi: 10.1016/j.gpb.2018.12.012 (PMC8242268; doi:10.1016/j.gpb.2018.12.012)
Supplement: Supplementary Table S7 — The upregulated ratios of Sja MMA transcripts under stress conditions based on ddPCR and RNA-seqanalyses [file mmc7.docx]

**Table S7 The upregulated ratios of *Sja* MMA transcripts under stress conditions based on ddPCR and RNA-seq analyses**

|  | *SjaM1PDH* | |  | *SjaM1Pase* | |  | *SjaM2DH* |  | *SjaHK* | |
| --- | --- | --- | --- | --- | --- | --- | --- | --- | --- | --- |
|  | ***SjaM1PDH1*** | ***SjaM1PDH2*** |  | ***SjaM1Pase1*** | ***SjaM1Pase2*** |  |  |  | ***SjaHK1*** | ***SjaHK2*** |
| ddPCR analysis |  |  |  |  |  |  |  |  |  |  |
| Hyposaline/Control (female gametophyte) | 3.97 | 3.09 |  | 7.84 | 4.15 |  | 2.90 |  | 2.04 | 2.73 |
| Hyperthermia/Control (female gametophyte) | 1.30 | 1.05 |  | 1.34 | 3.27 |  | 1.98 |  | 1.50 | 1.45 |
| RNA-seq analysis |  |  |  |  |  |  |  |  |  |  |
| Hyposaline/Control (female gametophyte) | 3.75 | 1.20 |  | 1.91 | 2.00 |  | 16.19 |  | 4.63 | 6.60 |
| Hyperthermia/Control (female gametophyte) | 1.87 | 7.86 |  | 2.74 | 1.20 |  | 12.50 |  | 1.33 | 2.33 |
