# Supplementary material for: Transcriptomic and Proteomic Analysis of Mannitol-metabolism-associated Genes in Saccharina japonica
Source: Genomics Proteomics Bioinformatics. 2020 Nov 25;18(4):415–29. doi: 10.1016/j.gpb.2018.12.012 (PMC8242268; doi:10.1016/j.gpb.2018.12.012)
Supplement: Supplementary Table S8 — Primers and probes used in droplet digitalPCR [file mmc8.docx]

**Table S8 Primers and probes used in droplet digital PCR**

| **Genes** | **Primers/Probes** | **Sequence** |
| --- | --- | --- |
| *SjaM1PDH1* | Primer-F | TAACGCACTACCGGAGGACC |
|  | Primer-R | CGTGATCGCATCCATCACC |
|  | Probe | CCAAGGGTGAGACAGATCAGTTCCA |
| *SjaM1PDH2* | Primer-F | GACAAGCCGTTCGAAGAGC |
|  | Primer-R | TTCCGCCTCCACGTTCACT |
|  | Probe | TGAGAAGAGTGGGCGAGATGGTGAC |
| *SjaM1Pase1* | Primer-F | GAACATGCTTGACGCGGTAG |
|  | Primer-R | ACTTCTCTGACGCCGTTGC |
|  | Probe | AAGTTCGGCCTGAAGAGCTTCGC |
| *SjaM1Pase2* | Primer-F | ACGTGAACAACGCCATGCT |
|  | Primer-R | TCTCAGTGTCGCCGATGGTA |
|  | Probe | AAGAAGCTTCTTCAGGAGGACTTCGG |
| *SjaM2DH* | Primer-F | ATCTAGAGGCGCACCAGGAG |
|  | Primer-R | GAGCGTATTGCGGCTTCAC |
|  | Probe | ACGTGAACCCTTACTCCGGTATGGC |
| *SjHK1* | Primer-F | GGAGGTGTGCAACGTGAAGTA |
|  | Primer-R | ACCGTTCGGTCGTTGACAGT |
|  | Probe | CCGCTAACTCGTTCGACCAGATCTA |
| *SjHK2* | Primer-F | ATGCAACTGGCGCAGAATG |
|  | Primer-R | CCTCGGCGAGTTCTCCACTA |
|  | Probe | ATTTAGCGCAGGAGTCGTCAACAAC |
